# Resolution of Regenerative Anemia, Thrombocytopenia, and Syncope After Cytoreduction of an Infiltrative Angiolipoma in a Dog: A Case Report

**DOI:** 10.3390/vetsci13090975

**Published:** 2026-09-17

**Authors:** Jaeyong Chung, Tae-Seong Moon, Hwi-Yool Kim

**Affiliations:** 1 Laboratory of Veterinary Surgery, College of Veterinary Medicine, Konkuk University, 120 Neungdong-ro, Gwangjin-gu, Seoul 05029, Republic of Korea; dvmjjyong@gmail.com; 2 EUM Animal Medical Center, 15 Dongtan-daero 6-gil, Hwaseong-si 18501, Republic of Korea; xotjd6990@naver.com

**Keywords:** canine, infiltrative angiolipoma, regenerative anemia, thrombocytopenia, syncope, cytoreductive surgery

## Abstract

Angiolipomas are uncommon benign mesenchymal neoplasms composed of mature adipocytes and proliferating vascular channels. In dogs, infiltrative angiolipomas are rarely reported and have primarily been described as localized soft-tissue masses. An 11-year-old, 10.8 kg, castrated male Japanese Spitz was referred for evaluation of regenerative anemia, mild thrombocytopenia, recurrent syncopal episodes, and a 5-year history of a slowly enlarging ventral thoracic mass. Preoperative hematological evaluation demonstrated anemia with a hematocrit of 25.4% and an increased absolute reticulocyte count of 122.7 K/µL, supporting a regenerative erythropoietic response. The platelet count was mildly decreased at 109 × 10^3^/µL. D-dimer concentration was increased (737.61 ng/mL; reference interval, 0–250 ng/mL), whereas prothrombin time (9.5 s; reference interval, 5–15 s) and activated partial thromboplastin time (15.6 s; reference interval, 15–45 s) were within their reference intervals. Referral history indicated that previous infectious and immune-mediated evaluations had not identified a definitive cause, although the original external testing records were unavailable for verification. Computed tomography revealed a heterogeneous subcutaneous mass with mixed fat- and soft-tissue-attenuating components, suspected infiltration of the superficial pectoral muscle, and focal rather than diffuse contrast enhancement. Surgical cytoreduction, including partial myectomy, was performed because complete excision was not considered feasible owing to deep muscular infiltration. Histopathological examination confirmed an infiltrative angiolipoma characterized by mature adipocytes, numerous vascular channels, skeletal muscle infiltration, and frequent intravascular fibrin thrombi. Following surgery, the hematological abnormalities progressively improved without blood transfusion or long-term immunosuppressive therapy. The platelet count increased from 109 × 10^3^/µL preoperatively to 153 × 10^3^/µL by postoperative day 16 and 202 × 10^3^/µL by postoperative day 56. During the same period, the hematocrit increased from 25.4% to 45.0%, and the absolute reticulocyte count subsequently returned to the reference interval. The syncopal episodes ceased after surgery and did not recur during the available 12-month follow-up. Fibrinogen concentration and peripheral blood smear evaluation were unavailable, and serial postoperative coagulation testing was not performed. Accordingly, the temporal association between tumor cytoreduction and hematological improvement raises the possibility of a tumor-associated process, but neither the underlying mechanism nor a causal relationship could be established.

## Simple Summary

Angiolipomas are uncommon benign tumors composed of fat cells and numerous blood vessels. In dogs, these tumors are rarely reported and usually cause only local problems. We describe an 11-year-old dog with a long-standing infiltrative angiolipoma of the ventral thorax, accompanied by regenerative anemia, mild thrombocytopenia, and recurrent episodes of syncope. Computed tomography showed that the mass extended into the underlying muscle, and histopathology confirmed an infiltrative angiolipoma containing numerous vascular channels and intravascular fibrin thrombi. Because complete removal was not possible, surgical cytoreduction was performed. Following surgery, the hematological abnormalities progressively improved, and the syncopal episodes did not recur during the available follow-up. Fibrinogen concentration was not measured and peripheral blood smear evaluation was unavailable; therefore, a consumptive or microangiopathic mechanism could not be confirmed. The temporal association between cytoreduction and clinical improvement suggests a possible tumor-associated process, but a causal relationship remains unproven.

## 1. Introduction

Angiolipomas are uncommon benign mesenchymal neoplasms characterized by an admixture of mature adipocytes and proliferating vascular channels [1]. Angiolipomas are generally classified as non-infiltrative or infiltrative according to their growth pattern. Non-infiltrative tumors are typically well circumscribed, whereas infiltrative angiolipomas extend into adjacent skeletal muscle or connective tissue and may therefore be difficult to excise completely [2,3]. In humans, intravascular fibrin thrombi have been described as a characteristic, although not pathognomonic, histological feature of angiolipoma [1,2]. Similar intravascular fibrin thrombi have also been documented in canine angiolipomatous tumors, including infiltrative angiolipoma [4,5]; however, their frequency and diagnostic significance in dogs have not been systematically established. In humans, these tumors most commonly occur in the subcutaneous tissues of the extremities and trunk and may be painful or multifocal [1,2].

Angiolipomas are rarely reported in veterinary medicine, and information regarding their clinical behavior and anatomical distribution remains limited [4,6]. In dogs, reported angiolipomas are generally solitary masses and appear to occur frequently in the trunk, although other anatomical locations have also been described, including the parotid region and spinal extradural space [5,6,7]. Unlike the painful or multifocal presentation commonly described in humans, canine lesions have more often been reported as painless, localized masses [4,6]. Infiltrative variants may extend into adjacent skeletal muscle and other soft tissues, complicating complete surgical excision [4,5,8]. Most previously reported canine cases have primarily been associated with local effects related to the size, location, or infiltrative behavior of the tumor, whereas systemic hematological abnormalities have not been emphasized as a major clinical feature.

The present report describes a dog with an infiltrative angiolipoma associated with regenerative anemia, thrombocytopenia, and recurrent syncope, followed by sustained hematological and clinical improvement after surgical cytoreduction. The purpose of this report is to expand the currently limited clinical information regarding canine infiltrative angiolipoma and to describe the temporal association between tumor cytoreduction and improvement in concurrent systemic hematological abnormalities. A tumor-associated thrombotic or consumptive process is considered only one possible, unconfirmed mechanism.

## 2. Case Presentation

### 2.1. Patient Information and Clinical History

An 11-year-old, 10.8 kg, castrated male Japanese Spitz was referred to the EUM Animal Medical Center for evaluation of regenerative anemia, mild thrombocytopenia, and recurrent episodes of syncope. The dog also had a long-standing subcutaneous mass in the ventral thoracic region. According to the owner, the mass had been present for approximately five years and had gradually increased in size. The syncopal episodes had begun approximately two weeks before referral and occurred approximately once daily on about three days per week. More detailed information regarding episode duration, specific triggers, relationship to exercise or feeding, posture, seizure-like activity, and recovery time was not consistently documented in the available record.

Prior to referral, diagnostic investigations had been performed at another veterinary hospital to determine the cause of the hematological abnormalities and recurrent syncope. Referral history indicated that infectious disease polymerase chain reaction testing, tick-borne disease screening, and evaluation for immune-mediated disease had been performed without clinically relevant abnormalities. However, the specific analytes tested, exact test dates, and original external laboratory reports were unavailable for independent verification. No definitive infectious, immune-mediated, or overt hemorrhagic etiology was identified based on the information available at referral. Bone marrow cytology or histopathology was not performed.

### 2.2. Physical Examination

On physical examination, the dog was quiet, alert, and responsive (QAR). Body temperature was 39.6 °C, heart rate was 140 beats/min, respiratory rate was 20 breaths/min, and blood pressure was 150 mmHg. The mucous membranes were pale pink, with a capillary refill time of <2 s. Cardiac auscultation revealed no audible murmur or pathologic arrhythmia and no other clinically significant abnormality.

An approximately 3 cm subcutaneous mass was palpated over the caudal ventral thorax, corresponding to the long-standing lesion described by the owner. The mass was somewhat mobile and nonpainful on palpation. Detailed assessment of its consistency and overlying skin involvement was limited on physical examination. No other clinically relevant abnormalities that could account for the recurrent syncopal episodes were identified on the available physical examination.

### 2.3. Laboratory Evaluation

A complete blood count was performed using an automated hematology analyzer (ProCyte Dx™, IDEXX Laboratories, Inc., Westbrook, ME, USA). D-dimer concentration was measured using a fluorescence immunoassay analyzer (Vet chroma™, ANIVET Diagnostics Inc., Chuncheon, Republic of Korea). Prothrombin time (PT) and activated partial thromboplastin time (aPTT) were measured using a point-of-care coagulation analyzer (qLabs^®^ Vet QV-3 Plus, Micropoint Biotechnologies Co., Ltd., Shenzhen, China).

Preoperative complete blood count revealed anemia, characterized by a decreased erythrocyte count (RBC, 4.35 × 10^12^/L), hemoglobin concentration (HGB, 8.8 g/dL), and hematocrit (HCT, 25.4%). Erythrocyte indices showed a mean corpuscular volume (MCV) of 58.4 fL, mean corpuscular hemoglobin (MCH) of 20.2 pg, mean corpuscular hemoglobin concentration (MCHC) of 34.6 g/dL, and red cell distribution width (RDW) of 19.4%. The absolute reticulocyte count was increased to 122.7 K/µL, supporting an active regenerative erythropoietic response. The preoperative anemia was microcytic, with MCV below the reference interval, whereas MCHC and RDW remained within their respective reference intervals. Thus, the regenerative classification was based primarily on the increased absolute reticulocyte count rather than on macrocytosis or increased RDW. Concurrent mild thrombocytopenia was present, with a platelet count of 109 × 10^3^/µL. Reference intervals for the hematological parameters are provided in Table 1.

Coagulation testing demonstrated an increased D-dimer concentration of 737.61 ng/mL (reference interval, 0–250 ng/mL), while PT (9.5 s; reference interval, 5–15 s) and aPTT (15.6 s; reference interval, 15–45 s) were within their respective reference intervals. The elevated D-dimer indicated increased fibrin formation and degradation but was nonspecific. In the absence of PT/aPTT prolongation and without a fibrinogen measurement, these findings were insufficient to establish a systemic consumptive coagulopathy.

Previous diagnostic investigations performed before referral reportedly included infectious disease polymerase chain reaction testing, tick-borne disease screening, and evaluation for immune-mediated disease, with no causative abnormality reported. However, the original external reports were unavailable; therefore, the exact tests and dates could not be independently verified. No overt internal hemorrhage was identified during the available diagnostic workup. Bone marrow examination was not performed; therefore, primary bone marrow disease could not be completely excluded.

### 2.4. Cardiovascular Evaluation

Cardiovascular evaluation was performed preoperatively to investigate a possible cardiac cause of the recurrent syncopal episodes. Resting electrocardiography revealed sinus arrhythmia and a deep S wave suggestive of mean electrical axis deviation; however, no clinically significant arrhythmia was documented. Continuous electrocardiographic monitoring during anesthesia and surgery also did not identify any arrhythmias.

Preoperative echocardiography revealed mild mitral regurgitation and mild diastolic dysfunction, without left atrial enlargement or objective evidence of right-sided heart failure. The N-terminal pro-B-type natriuretic peptide (NT-proBNP) concentration was increased to 1226 pmol/L (reference interval, 0–900 pmol/L). Although these findings indicated mild cardiac abnormalities, they were not considered sufficient to explain the recurrent syncopal episodes.

Based on the available cardiovascular evaluation, a primary cardiogenic cause of syncope was considered less likely. However, because ambulatory 24-h Holter monitoring was not performed, intermittent arrhythmia could not be completely excluded as a cause of the syncopal episodes.

### 2.5. Diagnostic Imaging

Preoperative whole-body computed tomography (CT; 256-slice Aquilion, Toshiba Medical Systems, Otawara, Japan) was performed to evaluate the local extent of the ventral thoracic mass and to screen for concurrent systemic disease or distant metastasis. For retrospective quantitative assessment of attenuation, circular regions of interest (ROIs) were placed on matched precontrast and two sequential post-contrast series. A 0.896 cm^2^ ROI was placed within a representative region of the mass, and a smaller 0.0704 cm^2^ ROI was placed over a focal intralesional vascular-appearing region. The same ROI size and anatomical location were maintained across the corresponding series where feasible. Because the exact contrast-injection time could not be recovered retrospectively, the two enhanced series were designated as the first and second post-contrast acquisitions rather than assigned to specific vascular phases.

**Figure 1:**
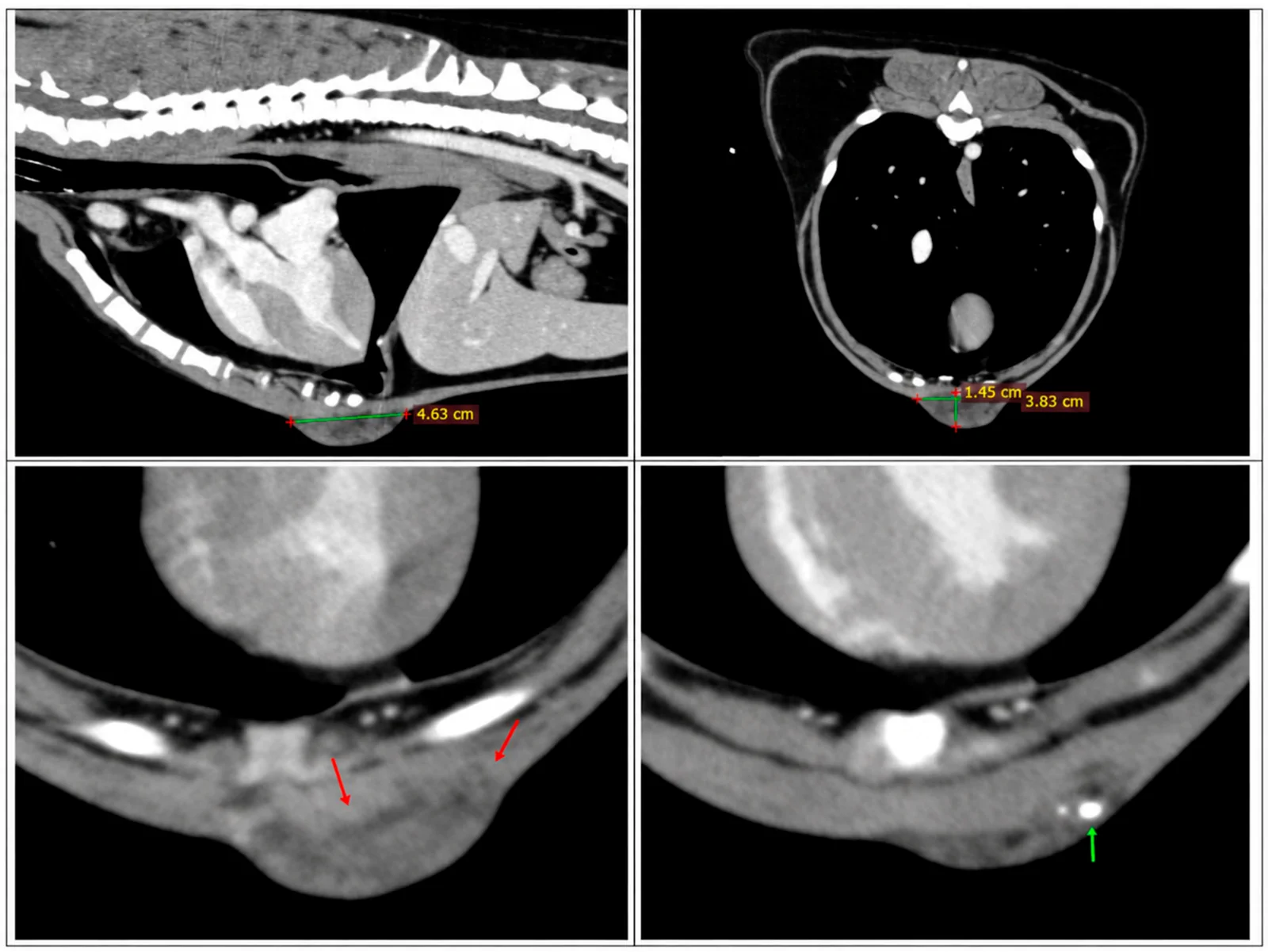
Computed tomography images of the ventral thoracic mass. Sagittal and transverse post-contrast CT images demonstrate a heterogeneous subcutaneous mass measuring approximately 4.6 × 3.8 × 1.5 cm at the level of the caudal sternum, with intermixed fat- and soft-tissue-attenuating components and focal contrast-enhancing regions. Mild infiltration into the underlying superficial pectoral muscle is suspected (red arrows), and a focal area of dystrophic calcification is present (green arrow). Quantitative attenuation measurements from the precontrast and sequential post-contrast series are described in Section 2.5.

CT revealed a subcutaneous mass measuring approximately 4.6 × 3.8 × 1.5 cm at the level of the caudal sternum. The lesion was heterogeneous, with intermixed fat- and soft-tissue-attenuating components. Retrospective quantitative analysis of the representative mass ROI yielded mean attenuation values of 10.2 HU on precontrast images, 14.3 HU on the first post-contrast acquisition, and 7.9 HU on the second post-contrast acquisition, indicating no substantial diffuse enhancement of the sampled mass tissue. In contrast, the focal intralesional vascular-appearing ROI showed mean attenuation values of 0.7 HU, 1.3 HU, and 29.4 HU on the precontrast, first post-contrast, and second post-contrast acquisitions, respectively. The corresponding maximum attenuation values increased from 27 HU precontrast to 40 HU and 102 HU on the first and second post-contrast acquisitions. These findings supported focal, rather than diffuse, contrast enhancement within the heterogeneous lesion. Mild infiltration into the underlying superficial pectoral muscle was suspected, and a focal area of dystrophic calcification was also identified (Figure 1). Based on the mixed fat-containing and soft-tissue components and the infiltrative appearance, an infiltrative adipocytic tumor was considered most likely, although other soft-tissue neoplasms, including liposarcoma, could not be excluded on CT alone.

No evidence of pulmonary or distant metastasis was identified. No other major abnormalities were detected on whole-body CT that could provide an alternative explanation for the recurrent syncopal episodes.

### 2.6. Surgical Treatment and Histopathological Findings

**Figure 2:**
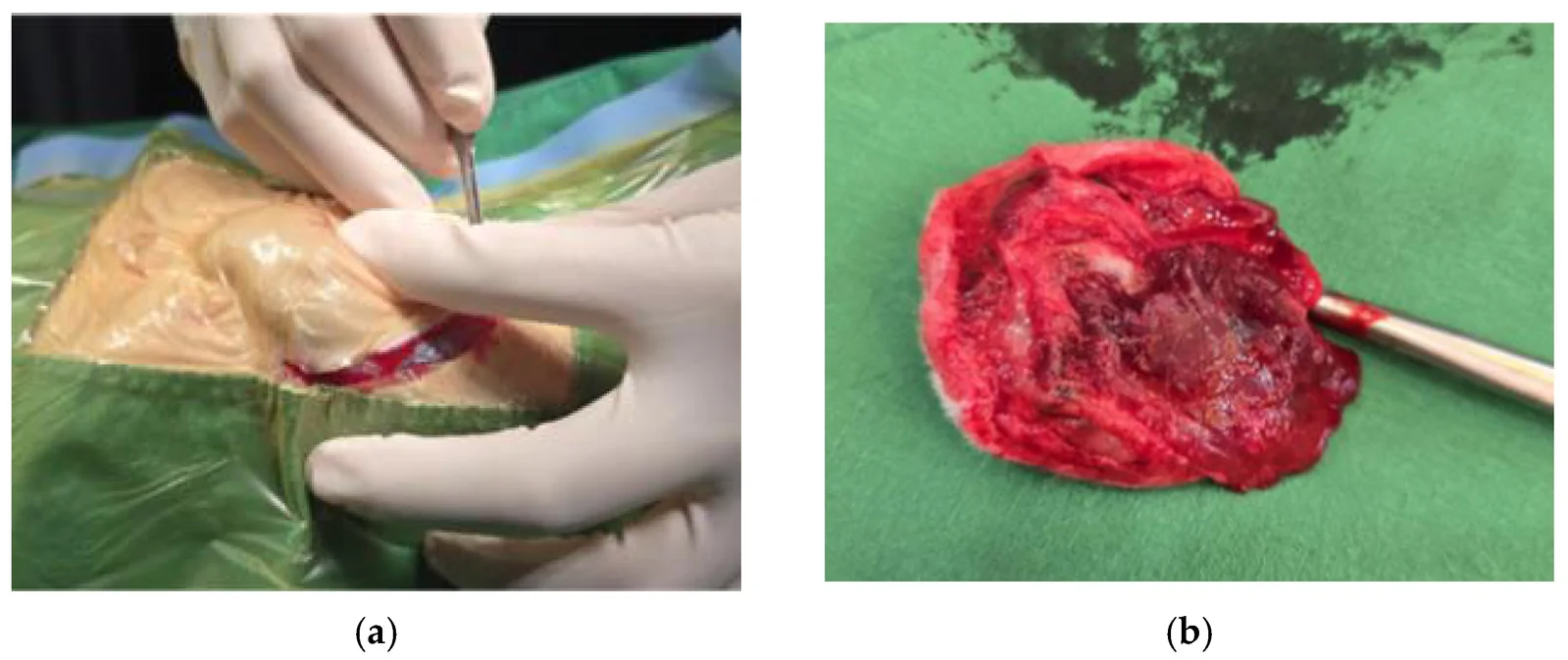
Intraoperative and gross appearances of the ventral thoracic mass. (**a**) Intraoperative photograph showing the highly vascularized mass firmly adhered to the underlying superficial pectoral muscle. (**b**) Gross appearance of the excised specimen following cytoreduction and partial myectomy.

Based on the clinical and imaging findings, surgical cytoreduction was performed for both diagnostic and therapeutic purposes. Intraoperatively, the mass was highly vascularized and firmly adhered to the underlying superficial pectoral muscle (Figure 2a). Because the lesion extended into the adjacent musculature, cytoreduction was combined with partial myectomy of the involved superficial pectoral muscle to achieve maximal removal of the grossly visible tumor (Figure 2b). Complete excision was not considered feasible because of the deep and diffuse muscular infiltration.

**Figure 3:**
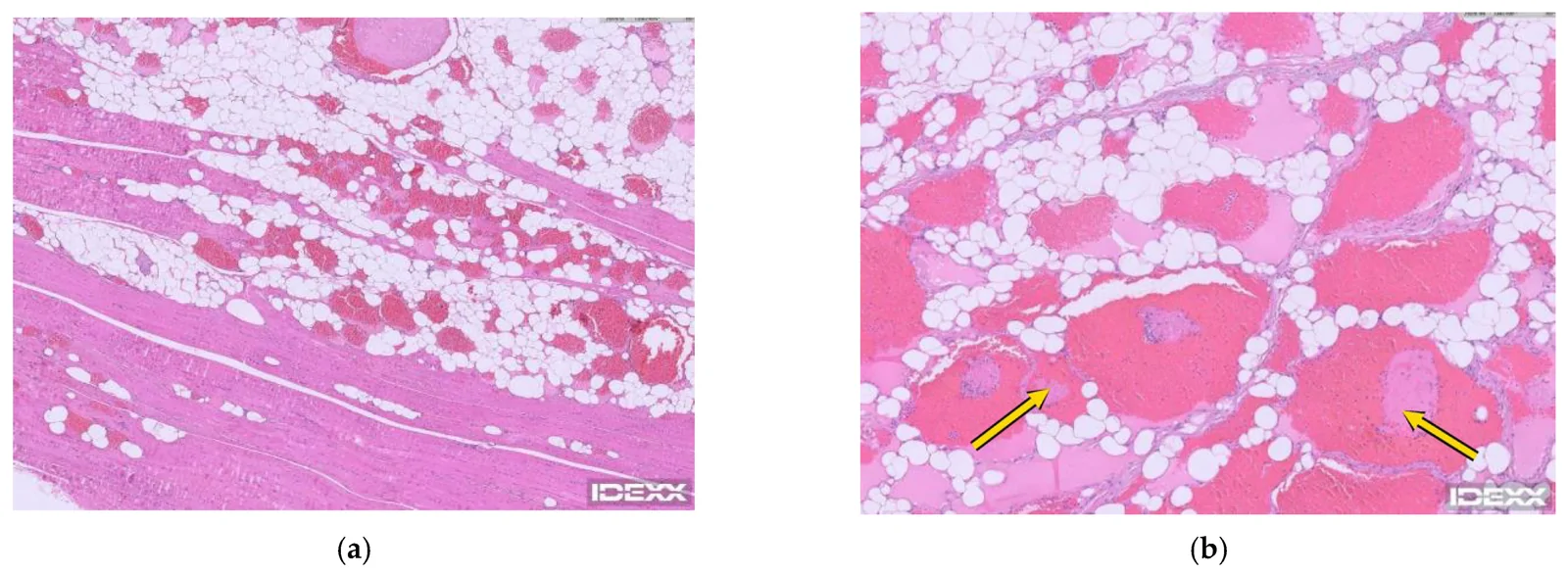
Histopathological findings of the infiltrative angiolipoma. (**a**) The mass was composed of mature adipocytes admixed with numerous vascular channels, with infiltrative extension into adjacent skeletal muscle. H&E, ×100. (**b**) Higher-magnification image showing vascular channels containing erythrocytes and intravascular fibrin thrombi (yellow arrows). H&E, ×400.

The excised tissue was submitted for histopathological examination (IDEXX Laboratories, Seoul, Republic of Korea). Histologically, the mass consisted of mature adipocytes interspersed with numerous thin-walled vascular channels of varying calibers (Figure 3a,b). The adipocytic and vascular components extended between and separated fascicles of the underlying skeletal muscle, confirming the infiltrative growth pattern. Frequent intravascular fibrin thrombi were identified within the vascular spaces. At the deep surgical margin, lesional tissue extended into the skeletal muscle, confirming incomplete histological excision.

No marked cellular atypia, malignant endothelial proliferation, or significant mitotic activity was identified. Based on the combined histopathological features, the lesion was diagnosed as an infiltrative angiolipoma rather than a malignant vascular neoplasm.

### 2.7. Postoperative Course and Clinical Outcomes

Following surgical cytoreduction, the dog showed progressive improvement in the hematological abnormalities without blood transfusion or long-term immunosuppressive therapy. Postoperative supportive treatment consisted of intravenous cefazolin (20 mg/kg), famotidine (0.5 mg/kg), tramadol (1.5 mg/kg), and routine crystalloid fluid therapy with Hartmann’s solution. No corticosteroid, iron supplementation, anticoagulant, antiplatelet, or other treatment specifically directed at the anemia or thrombocytopenia was administered. Serial changes in erythrocyte parameters, absolute reticulocyte count, and platelet count are summarized in Table 1.

The erythrocyte count progressively increased from 4.35 × 10^12^/L preoperatively to 4.45 × 10^12^/L on postoperative day (POD) 3, 5.05 × 10^12^/L on POD 16, and 6.80 × 10^12^/L on POD 56. Hemoglobin concentration showed a similar improvement, increasing from 8.8 g/dL preoperatively to 9.3 g/dL on POD 3, 10.8 g/dL on POD 16, and 15.1 g/dL on POD 56. The hematocrit increased from 25.4% preoperatively to 27.5%, 32.4%, and 45.0% on PODs 3, 16, and 56, respectively.

The mean corpuscular volume increased from 58.4 fL preoperatively to 61.8 fL on POD 3 and 64.2 fL on POD 16, reaching 66.2 fL by POD 56. The mean corpuscular hemoglobin increased concurrently from 20.2 pg to 20.9, 21.4, and 22.2 pg, respectively. Mean corpuscular hemoglobin concentration remained relatively stable throughout follow-up, ranging from 33.3 to 34.6 g/dL. Red cell distribution width increased numerically from 19.4% preoperatively to 20.5% on POD 3 and 21.3% on POD 16 while remaining within the reference interval, and subsequently decreased to 18.0% by POD 56.

The absolute reticulocyte count increased from 122.7 K/µL preoperatively to 198.9 K/µL on POD 3 and remained increased at 198.1 K/µL on POD 16, indicating a sustained regenerative erythropoietic response during recovery from anemia. By POD 56, the reticulocyte count had decreased to 66.6 K/µL as the erythrocyte count, hemoglobin concentration, and hematocrit normalized.

The platelet count also progressively increased from 109 × 10^3^/µL preoperatively to 127 × 10^3^/µL on POD 3, 153 × 10^3^/µL on POD 16, and 202 × 10^3^/µL on POD 56. Thus, the thrombocytopenia had resolved by POD 16 and remained within the reference interval thereafter.

The recurrent syncopal episodes ceased following surgery. Body weight was 10.8 kg at initial presentation and 11.65 kg on the day of surgery; body weight at subsequent follow-up visits was 11.25 kg and 11.2 kg, respectively. Appetite and general activity were reported to be very good throughout the documented follow-up period. At the final in-person recheck, corresponding to the POD 56 hematology assessment reported above, no clinically apparent local recurrence or other new abnormality was identified. At a 12-month telephone follow-up, the owner reported no recurrence of syncope, no clinically apparent mass regrowth, and no other clinical concerns. Further follow-up could not be obtained because subsequent telephone contact with the owner was unsuccessful. No standardized systemic functional assessment beyond serial body weight and owner-reported appetite and activity was performed.

## 3. Discussion

This case describes an infiltrative angiolipoma in a dog associated with regenerative anemia, mild thrombocytopenia, and recurrent syncope, followed by progressive hematological improvement and cessation of syncopal episodes after surgical cytoreduction. Canine angiolipomas are rarely reported, and previously described cases have predominantly presented as localized masses associated with infiltration, compression, or other regional effects [4,5,6,7,8]. Systemic hematological abnormalities have not been emphasized as a characteristic clinical feature in these reports. Therefore, the present case expands the clinical spectrum of canine infiltrative angiolipoma by describing concurrent hematological abnormalities and their temporal improvement following tumor cytoreduction, without establishing a direct causal relationship.

The complete hematological evaluation further characterized the anemia. Preoperatively, the dog had microcytic anemia with an increased absolute reticulocyte count, whereas MCHC and RDW remained within their reference intervals. During postoperative recovery, erythrocyte count, hemoglobin concentration, and hematocrit progressively normalized, while MCV increased into the reference interval. RDW showed only modest numerical variation and remained within the reference interval throughout follow-up. Therefore, the regenerative nature of the anemia was supported primarily by the increased absolute reticulocyte count rather than by macrocytosis or an increased RDW. Serum iron, total iron-binding capacity, and ferritin concentrations were not measured. Iron studies were not included in the original clinical diagnostic workup, and because this is a retrospective case report, the missing baseline measurements could not be obtained after the fact. Consequently, iron deficiency, including that associated with chronic occult blood loss, could not be excluded as a contributor to the microcytosis. Unrecognized hemolysis or a mixed mechanism could also have coexisted with a tumor-associated process. The available data do not allow the relative contribution of these alternative mechanisms to be determined.

A localized tumor-associated thrombotic or consumptive process was considered one possible explanation for the concurrent hematological abnormalities, but the evidence was incomplete. Histopathological examination demonstrated numerous vascular channels containing frequent intravascular fibrin thrombi, and laboratory testing showed mild thrombocytopenia and an increased D-dimer concentration. However, PT and aPTT were within their reference intervals, fibrinogen concentration was not measured, and postoperative D-dimer, PT, and aPTT measurements were not available. Accordingly, the available findings raise the possibility of a localized thrombotic process but do not demonstrate systemic consumptive coagulopathy. Mechanical erythrocyte injury within the abnormal tumor vascular bed was also considered possible; however, a microangiopathic hemolytic process could not be demonstrated because peripheral blood smear evaluation for schistocytes was unavailable.

Comparable hemostatic abnormalities have been reported in dogs with other vascular neoplasms. In a prospective study of 24 dogs with histologically confirmed hemangiosarcoma, thrombocytopenia was present in 75%, and 50% met the study criteria for disseminated intravascular coagulation at presentation [9]. In a retrospective study of dogs with cutaneous hemangiomas and hemangiosarcomas, tumor-associated hemostatic defects included hemorrhage, thrombocytopenia, hypofibrinogenemia, and findings associated with disseminated intravascular coagulation [10]. These reports demonstrate that canine vascular neoplasms can be accompanied by systemic hemostatic abnormalities and provide biological context for the present case. However, hemangiosarcoma and hemangioma are biologically distinct from benign infiltrative angiolipoma, and direct extrapolation of their mechanisms or clinical behavior to the present case is not possible.

This proposed mechanism has some conceptual similarities to the Kasabach–Merritt phenomenon (KMP), a consumptive coagulopathy associated with vascular tumors and characterized by thrombocytopenia, coagulation abnormalities, and hypofibrinogenemia [11]. A human case of infiltrative angiolipoma associated with Kasabach–Merritt syndrome has previously been reported [12], providing a conceptual precedent for tumor-associated consumption in an angiolipoma. However, the thrombocytopenia in the present dog was mild, PT and aPTT were within their reference intervals, and fibrinogen concentration was unavailable. Therefore, the present findings should not be interpreted as evidence of true KMP; rather, KMP serves only as a comparative model for a possible localized thrombotic or consumptive process associated with the abnormal vascular architecture of the tumor.

The postoperative course provides evidence of a temporal association between tumor cytoreduction and hematological improvement. Following cytoreduction, the erythrocyte count, hemoglobin concentration, hematocrit, and platelet count progressively returned to their respective reference intervals without blood transfusion, corticosteroid treatment, iron supplementation, anticoagulant therapy, or long-term immunosuppressive therapy, while the reticulocyte count normalized as the anemia resolved. The recurrent syncopal episodes also ceased after surgery and were not reported during the available 12-month follow-up. Although preoperative electrocardiography and echocardiography did not identify abnormalities considered sufficient to explain the episodes, intermittent arrhythmia could not be completely excluded because 24-h Holter monitoring was not performed. Thus, the absence of recurrent syncope after surgery represents a temporal association and should not be interpreted as proof that the tumor was the cause of the episodes. Although no treatment specifically directed at the anemia or thrombocytopenia was administered, spontaneous improvement, nonspecific effects of perioperative fluid therapy, or other unmeasured perioperative factors cannot be completely excluded as contributors to the postoperative changes.

Canine infiltrative angiolipomas are histologically benign but locally aggressive and may be difficult to excise completely because of infiltration into adjacent tissues [5,8]. In a previously reported canine parotid infiltrative angiolipoma for which further surgery was declined, the mass increased in size by approximately 30% during 14 months of follow-up despite the dog remaining clinically well [5]. These limited case-based data indicate that residual lesions may persist or slowly regrow. Accordingly, long-term clinical surveillance of the surgical site, with repeat imaging when local regrowth is suspected, is appropriate after cytoreductive or incomplete excision. In the present dog, no clinically apparent local regrowth was identified at the POD 56 recheck or reported by the owner at the 12-month telephone follow-up; however, longer-term objective surveillance was not available.

Several limitations should be considered. Peripheral blood smear evaluation was unavailable, preventing assessment for schistocytes or other morphological evidence of erythrocyte injury, and fibrinogen concentration was not measured, limiting characterization of the proposed thrombotic or consumptive process. Serial postoperative D-dimer, PT, and aPTT measurements were not obtained, and residual tumor volume was not quantitatively assessed by follow-up imaging; therefore, a direct relationship between tumor burden, coagulation activity, and hematological recovery could not be demonstrated. Iron studies were not performed, so iron deficiency or chronic occult blood loss could not be excluded. Original records from the pre-referral infectious and immune-mediated evaluations were unavailable, preventing verification of the exact tests and dates. Bone marrow examination was not performed. Holter monitoring and advanced neurological evaluation were also not performed to fully investigate alternative causes of syncope. Although serial body weights and owner-reported appetite and activity were available, no standardized postoperative functional or systemic outcome assessment was performed. Objective in-person follow-up was limited to POD 56, with a subsequent owner telephone update at 12 months; further follow-up could not be obtained. Finally, this report describes a single patient and therefore cannot establish a causal relationship or determine whether similar hematological abnormalities occur in other dogs with angiolipoma.

## 4. Conclusions

This case describes a canine infiltrative angiolipoma associated with regenerative anemia, mild thrombocytopenia, and recurrent syncope, all of which improved or resolved following surgical cytoreduction. The temporal relationship between cytoreduction and hematological recovery raises the possibility that the tumor contributed to the systemic abnormalities; however, the underlying mechanism remains uncertain, and a causal relationship cannot be established from this single case. Further clinical reports are needed to determine the mechanism and clinical significance of this association in dogs.

## Figures and Tables

**Table 1:** Serial changes in hematological parameters before and after surgical cytoreduction.

Parameter	Preoperative (Day 0)	POD 3	POD 16	POD 56	Reference Interval
RBC (×10^12^/L)	4.35	4.45	5.05	6.80	5.65–8.87
HGB (g/dL)	8.8	9.3	10.8	15.1	13.1–20.5
HCT (%)	25.4	27.5	32.4	45.0	37.3–61.7
MCV (fL)	58.4	61.8	64.2	66.2	61.6–73.5
MCH (pg)	20.2	20.9	21.4	22.2	21.2–25.9
MCHC (g/dL)	34.6	33.8	33.3	33.6	32.0–37.9
RDW (%)	19.4	20.5	21.3	18.0	13.6–21.7
Absolute reticulocytes (K/µL)	122.7	198.9	198.1	66.6	10.0–110.0
Platelets (×10^3^/µL)	109	127	153	202	148–484

Abbreviations: RBC, red blood cell count; HGB, hemoglobin; HCT, hematocrit; MCV, mean corpuscular volume; MCH, mean corpuscular hemoglobin; MCHC, mean corpuscular hemoglobin concentration; RDW, red cell distribution width; POD, postoperative day.

## Data Availability

The raw data supporting the conclusions of this article will be made available by the authors on request.
